# Influence of digital media in the oral health education of mother-child pairs: study protocol of a parallel double-blind randomized clinical trial

**DOI:** 10.1186/s13063-022-06602-4

**Published:** 2022-08-09

**Authors:** Yuri Jivago Silva Ribeiro, Luanna Gonçalves Ferreira, Paulo Nelson-Filho, Maya Fernanda Manfrin Arnez, Francisco Wanderley Garcia Paula-Silva

**Affiliations:** Department of Pediatric Dentistry, School of Dentistry of Ribeirão Preto, University of São Paulo, Avenida do Café, s/n. CEP 14040-904, Bloco M, Sala 28, São Paulo, Ribeirão Preto, SP Brazil

**Keywords:** Dental caries, Oral health, Digital media, Mother, Child

## Abstract

**Background:**

Dental caries is the most common non transmissible chronic disease in childhood and the control of dental biofilm in children is one of the greatest challenges in oral disease prevention. Digital media applications can help patients in improving their oral hygiene performance and reducing the number of appointments due to pain and discomfort reasons. This study aims to investigate the use of an smartphone application (WhatsApp) to deliver oral health education to mother-child pairs, with the ultimate goal of controlling dental biofilm and caries through digital activities focused on oral hygiene.

**Methods:**

This study was designed as a randomized, double-blind, parallel clinical trial involving 100 pairs of mothers and children (6–12 years old). The mothers and children will be randomly allocated to the control group (*n* = 50 pairs), who will receive a single visit conventional oral health education, or to the experimental group (*n* = 50 pairs), who will receive both a single visit conventional oral health education and educational videos through WhatsApp Messenger, twice a week. Before randomization of the groups and after the intervention, pairs will be evaluated regarding to plaque index (VPI), gingival bleeding index (GBI), and number of decayed, missing and filled permanent or primary teeth (DMF-T) modified by the inclusion of active non-cavitated carious lesions (Nyvad criteria). Socioeconomic data, dental history, and oral health literacy will obtained using questionnaires (Oral Health Literacy Assessment Task for Paediatric Dentistry; BOHLAT-P). Chi-square, Student’s *t*-test, paired Student’s *t*-test, Mann-Whitney, and Friedman tests will be used with a 5% significance level.

**Discussion:**

This intervention proposal is designed to motivate behavioral change in mother-child pairs. We hypothesize that adding digital media to traditional oral health programs will provoke improvements in oral hygiene behavior and health outcomes. To our knowledge, this is the first study evaluating the effect of educational videos communicated by digital media (WhatsApp) on the oral health of mother-child pairs evaluated by long-term dental examinations. In addition, we will assess the maternal level of comprehension of the provided information via a literacy assessment tool. The clinical trial is registered at the Brazilian Registry of Clinical Trials (No. RBR-7s8bw6m).

**Supplementary Information:**

The online version contains supplementary material available at 10.1186/s13063-022-06602-4.

## Background

Dental caries is the most common non transmissible chronic disease in childhood and a major public health problem worldwide [1–3]. One of the greatest challenges in oral disease prevention is the control of dental biofilms and gingival inflammation in children [4, 5]. Because prevention plays a crucial role in reducing oral health problems [6], adequate oral care, including dental flossing, brushing, and diet control (in particular, reducing sugar intake) should be initiated on the eruption of the first teeth, as an effective strategy to prevent oral diseases [5, 7–9]. Early education is the basis for desirable habits and behaviors as well as for effective lifestyle changes in the future [10].

Dentists and dental teams are critical for early oral health education. Because they have theoretical and practical knowledge as well as the required training, dental teams play an essential motivational role in the practice and maintenance of good oral hygiene and the modification of poor habits [11–13]. The World Health Organization recommends that interventions involving oral health education be associated with other health promotional activities, particularly the development of healthy habits [14]. The existing literature suggests various methods for the promotion of oral health, education, and motivation including direct orientation, videos, leaflets, substances evidencing bacterial plaque, lectures, puppets, gymkhanas, theater, and music, among others [15–17].

Digital media such as the Internet, together with health organizations, also plays an important role in providing education, motivation, and information [18]. Since 2014, more than 50% of adults have searched for health information on the Internet [19]. Also,  previous study demonstrated that between 2016 and 2018, more than half of adults (50.4%) and children (58.8%) accessed primary dental care information via the Internet [20]. With the advancement of the Internet, numerous social media platforms have become available for personal interaction and information searches. To date, social media platforms have been used more frequently than traditional search engines [21].

Dental studies have achieved good results using an oral health education tool to inform and motivate adolescents [22, 23]. A digital medium with widespread use in Brazil is WhatsApp Messenger, which is an application that allows rapid interaction through texts, images, voice and video calls [24]. Consequently, this application may be an effective social media tool for disseminating health education [25]. In this context, a 2016 survey conducted in Brazil showed that 76% of users who searched the Internet made continuous use of WhatsApp, and this percentage is one of the highest worldwide, second only to South Africa [26]. However, the efficacy of using WhatsApp to promote oral health education for mothers and children has not yet been elucidated in the literature.

The objective of this parallel, double-blind, and randomized clinical trial is to compare the influence of conventional education promoting oral health, versus that of conventional education plus the WhatsApp digital platform, in controlling dental biofilm and caries in mother-child pairs. Mothers and their children (age 6–12 years) will be evaluated through a self-administered questionnaire on oral hygiene habits, and through the Oral Health Literacy Assessment Task for Paediatric Dentistry (BOHLAT-P). In addition, clinical parameters of the mother-child pairs (including the visible plaque index [VPI], gingival bleeding index [GBI], number of decayed, missing, and filled deciduous teeth [dmft], and the numbers of decayed, missing, and filled permanent teeth [DMFT modified by the inclusion of active non-cavitated carious lesions]) will be considered in the assessment. We hypothesized that mother-child pairs randomized to the experimental group would exhibit greater improvements in oral hygiene behavior and health outcomes when compared with the control group during follow-up assessments at 1, 6, and 12 months.

## Materials and methods

### Ethics approval and consent to participate

This clinical trial was approved by the Committee for Ethics in Research Involving Human Beings (CAAE: 50783521.9.0000.5419) and is outlined according to CONSORT recommendations. The trial has been registered with the Brazilian Registry of Clinical Trials (# RBR-7s8bw6m).

### Study design

This study is a randomized, double-blind, parallel clinical trial with voluntary participation and 12 months of follow-up. The protocol follows the recommendations of the 2013 interventional trials (SPIRIT) guidelines.

### Sample size and power calculation

To calculate the sample size, we used the study by Zolfaghari et al. [10], which measured the efficacy of a game for delivering oral health knowledge to children and mothers. Considering an alpha value of 5% and a test power of 80%, the sample calculation requirement was 44 mother-child pairs per group (control and experimental).

### Participants and study setting

After anamnesis and initial clinical examination, the dental examination will be performed at the Clinical Research Center at the School of Dentistry of Ribeirão Preto at University of São Paulo, Brazil (FORP/USP). Once the study details are explained and queries are addressed, volunteers (mothers) are expected to sign the informed consent form (TCLE), and the children are expected to sign the assent form.

### Inclusion criteria

All mothers have to be at least 18 years old

Children (6–12 years old) of both sexes

Participants (mother-child pairs) with good general health (reported by the mother)

Mothers using WhatsApp Messenger on their smartphones

### Exclusion criteria

Participants (mothers and children) exhibiting learning difficulties

Mothers with total upper and lower dental prostheses

### Interventions

The following study groups will be created in parallel to evaluate the proposed intervention. (1) Control group: Both mothers and children will undergo clinical examination, prophylaxis, topical fluoride application, and oral hygiene guidance on a single dental office visit. The pairs will receive an oral hygiene in-person orientation session that will take about 30 min regarding to oral health, gingival health, sugar intake, fluoride importance in prevention of dental caries, and oral hygiene habits, complemented with illustrative resources.

(2) Experimental group: Both mothers and children will receive clinical examinations, prophylaxis, topical fluoride application, and oral hygiene guidance on a dental office visit. Additionally, the digital platform WhatsApp Messenger (Mountain View, California) will be used to provide educational videos to the mother-child pairs, twice per week (Tuesday and Thursday) at 7 PM over a period of 4 weeks. A WhatsApp video presenting a story on oral health, along with a reminder to perform their oral hygiene routine (toothbrushing and flossing) will be sent to the mother-child pairs in the experimental group. Topics including oral hygiene, dental biofilms, white spot lesions, dental caries, and the importance of dental appointments will be covered. Videos were validated by educators and a group mother-child pairs who evaluated the quality of the animated videos and understanding of the topics covered according to Marinho et al. [27].

At the end of 4 weeks (T1), 6 months (T2), and 12 months (T3), the mother-child pairs will undergo follow-up clinical evaluations at FORP/USP. If needed, dental treatment will be performed by a research team at the same institution.

Theoretical training took place for an examiner (Y.J.S.R) along with the reference standard examiner (F.W.G.P.S) through expository classes regarding the following indexes: VPI; GBI; decayed, missing, and filled permanent teeth (DMFT); and decayed, missing, and filled primary teeth (dmft) and Nyvad criteria to assess active non-cavitated carious lesions. Oral examination of the mother-child pairs will be performed by a trained and calibrated operator (Y.J.S.R) and assisted by a trained annotator (L.G.F). The examinations will be performed with the mother-child pairs seated in front of the examiner. The dentist will be used individual protection equipment, a sterilized flat oral mirror (Golgran, São Caetano do Sul, São Paulo, Brazil), and a sterilized periodontal probe (WHO-621, Trinity®, Campo Mourão, PA, Brazil). The visible plaque index (VPI) will be used to evaluate the mesial, distal, vestibular, and lingual surfaces of the incisors and first upper and lower molars according to the criteria set by Ainamo and Bay [28]. Evaluations will be performed by direct visualization of the vestibular and palatine/lingual surfaces. A flat oral mirror # 5 (Golgran, São Caetano do Sul, São Paulo, Brazil) will be used for the visual examination. The established scores for the VPI are (1) presence and (0) absence. For the gingival bleeding index (GBI), a periodontal probe (WHO-621, Trinity®, Campo Mourão, PA, Brazil) will be used to evaluate the subgingival surfaces 1 mm into the sulcular epithelium. The bleeding on probing index will also be recorded as (1) presence of bleeding and (0) absence of bleeding. Oral hygiene will be considered unsatisfactory if the percentage of visible biofilm is greater than 15% and/or gingivitis is present, as indicated by bleeding at more than 15% of the evaluated sites [25, 28].

After brushing, a clinical oral examination will be performed to verify the participants’ oral health condition, and the data will be recorded using the dmft and DMFT index for deciduous and permanent teeth, respectively [29, 30]. Dental caries will be evaluated using the Nyvad Criteria to assess severity and activity of carious lesions, including lesions of rough and opaque white spots [31]. The initial evaluation aims to verify the inclusion criteria for the study participants. Subsequent evaluations will aim to assess the effectiveness of the interventions for improving the oral hygiene of the mother-child pairs.

### Randomization and allocation concealment

Patients will be randomized using a simple draw. A number will be assigned to each mother-child pair. A virtual randomization program [32] will be used to generate the allocation sequence in the groups, and the names of the mother-child pairs of the randomized groups (control and experimental) will be recorded by L.G.F. so that the clinical examiner (Y.J.S.R.) and the statistical evaluator (F.W.G.P.S.) will not have access to this information. L.G.F. will also be responsible for sending the video individually to each participant of the experimental group using the WhatsApp application. The participants will be blinded to the data collection. Both the examiner involved in the intervention and the statistician who will analyze the data through appropriate statistical tests will be blinded.

**Fig. 1:**
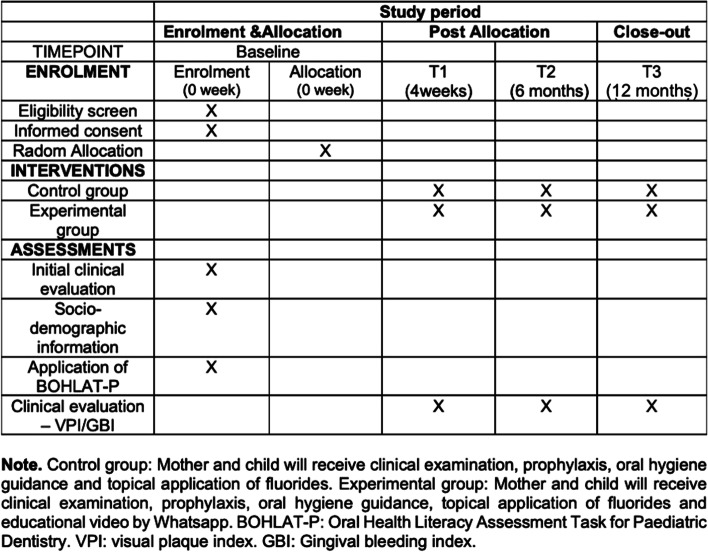
Schedule of enrollment, interventions, and assessments in the study

Figure 1 summarizes the study design, randomization process, group allocation, and evaluation periods.

### Questionnaires

A literacy assessment instrument (BOHLAT-P), validated for Brazilian Portuguese language, will be used to evaluate the mothers’ oral health knowledge, textual understanding, and numerical skills [33, 34]. The first part evaluates basic knowledge in oral health, from images illustrating normal deciduous dentition and dental caries. The second part measures the numerical abilities of the individual, being represented by four groups of questions. The first refers to an appointment card already completed, questioning the date and time of the next appointment, with the contact number of the clinic. The second group of questions is related to a drug prescription, in which the respondent is asked about the date of validity of the medication presented, adequate dose interval, and duration of treatment. The third group of questions revolves around post-surgical instructions, which cover aspects such as warm diet introduction period, oral hygiene, and conduct facing complications. The fourth group of questions has as its central axis a dentifrice label, addressing points such as brushing frequency and ideal dentifrice amount. The last part of the instrument evaluates textual comprehension and vocabulary. It consists of a dialogue between the dentist and the child’s mother addressing the exchange of dentitions with blank passages to be completed from the answer options presented. Lastly, there is a brushing guide whose instructions need to be sorted correctly.

A self-report questionnaire containing a series of questions ordered in the form of questions, addressing issues such as identification, socioeconomic status, maternal and paternal education, occupation of the mother and person with higher family income, health conditions, access to health services, and oral health care measures will be applied before interventions [35, 36].

### Outcome measurements

Measurements will be taken at baseline, post-intervention (i.e., 30 days after completion of the intervention), and at the 6-month and 12-month follow-up points. All follow-up assessments will be conducted by a researcher blinded to the group allocation of the participants.

### Baseline data

Sociodemographic and socioeconomic data will be collected at baseline, including the participant’s age, gender, skin color, household income, and educational literacy level.

### Measurement of primary outcomes

The primary outcome is defined as the effectiveness of educational videos via WhatsApp Messenger for controlling dental biofilm and dental caries in mother-child pairs. This outcome will be achieved by evaluating the VPI, GBI, and dmft indexes for deciduous teeth, and the DMFT for permanent dentition. Dental caries will be evaluated using the Nyvad Criteria to assess severity and activity of carious lesions. These results will be compared with those of the control group throughout the follow-up.

### Measurement of secondary outcomes

The secondary outcome is defined as the participant responses to the questionnaire on socioeconomic data, oral hygiene, visits to the dentist, and acceptability of the animation by the pairs mother-child. At the end of T3, the mothers of the experimental group will be asked if the videos were watched at the moment they were sent or if they was watched in other times after the intervention. Question will include how often the videos were watched, which videos they and their children liked the most, and what subject was easier or more difficult to understand and suggestions for improvement. Quality of life will be evaluated using the BOHLAT-P questionnaire [33]. This questionnaire contains 49 questions and is divided into three domains: oral health knowledge, textual understanding, and numerical skills [34]. The mother will answer the BOHLAT-P questionnaire alone, and the researchers will provide help if requested.

### Data management

The data will be collected in standardized dental records identified by numbers (obtained after randomization) for each participant (mother-child). All data will be scanned and stored in a Microsoft Excel spreadsheet for further statistical analysis at the end of the study. Upon completion of the study, data will be made publicly available at the University of Sao Paulo institutional repository.

### Strategies for study retention

During the study period, participants from both groups (control and experimental) will receive a text message informing them of the day and time for the next dental evaluation. In the case of no attendance, calls will be made to the participant (mother) to reschedule the missed dental evaluation.

### Statistical analyses

Data will be analyzed using the GraphPad Prism 8.0 software (GraphPad Software Inc., San Diego, CA, USA). Descriptive analyses, including percentages, means, and standard deviations, will be reported. Answers to the BOHLAP-T questionnaire will be dichotomized into correct and wrong answers. Each correct answer will receive a score of 1, and each wrong answer will receive a score of 0. The maximum and minimum scores are 49 and 0, respectively. The chi-square test will be used to analyze categorical variables, the Mann-Whitney test will be used to compare the inter-group differences, and Friedman test will be used to calculate intra-group differences in relation to VP and GBI. The following tests will be used to analyze the experience of caries: *t* of “Student” and of chi-square. The significance level will be set at 5%.

### Dissemination policy

We intend to disseminate the methods and results of our study to the general public through social media, presentations at international congresses on corresponding areas of interest (for example, dentistry), and by submission of manuscripts describing our findings to appropriate scientific journals.

## Discussion

To the best of our knowledge, this study is the first randomized clinical trial to evaluate the effect of digital media (WhatsApp) on the oral health of mother-child pairs. We will test the digital media effect on mother-child pairs by sending WhatsApp educational videos to participants followed by dental examinations. In addition, we will gauge the maternal level of comprehension of the provided information via a literacy assessment tool.

Dental caries is considered a major public health problem [37–39] because it is a behavioral, avoidable, and multifactorial disease related to age that could last a lifetime without prevention [40]. According to the above information, academies and dental associations recommend visiting dentists from an early age [10]. However, parents often neglect this first contact [41]. The use of smartphone applications has led researchers to investigate the possibilities of using technology to improve infant-juvenile health care [25, 42, 43]. Considering their wide use and ease of access to multiple resources, digital platforms can be suited to health promotion by improving parental knowledge, especially that of mothers [44]. In addition, these digital tools can be used to stimulate healthy habits in relation to oral health [10]. It is known that parents are primarily responsible for the state of health and oral hygiene of their children. In addition, parents typically supervise their children’s oral hygiene behavior as they grow [45, 46].

The promotion of oral health is a field that can benefit from social media, through web-based social networks and mobile [47]. In developing countries, digital technologies, particularly smartphones, have been widely adopted [48]. This allowed society to overcome barriers related to health promotion allowing participation in the digital environment [48]. And the use of different social tools are widely used to promote oral and systemic health, since they have resources for exchange and participation in open access information, providing a channel for interactive conversations and allowing users to read subjects of their interest [46–48]. In addition, the fast and continuous communication between people from different cities and countries through social networks facilitates the search for various subjects to clarify questions based on alternative virtual resources [47].

Based on the available information regarding application of digital tools to improve several health areas (such as medicine and dentistry) [22, 49], we hypothesized that educational videos sent to mother-child dyads by the WhatsApp application could improve oral hygiene behavior and outcomes, particularly when compared with traditional health-promotion programs. This intervention proposal was designed to target factors for behavior motivation and modification in mother-child pairs. The results of our study will have implications for the control of biofilms, and consequently caries disease, in mother-child pairs since health prevention and educational programs should begin with regular consultations with a dentist. Additionally, epidemiological research on oral health should be conducted periodically.

## Trials status

The protocol version is 1.

The trial is still recruiting patients. The first patient was recruited in January 2022. The last patient is anticipated to be recruited in December 2022.

## Supplementary Information

**Additional file 1.**

## Data Availability

Not applicable

## Abbreviations

VPI: Visible plaque index
GBI: Gingival bleeding index
DMFT: Numbers of decayed, missing, and filled permanent teeth
Dmft: Numbers of decayed, missing, and filled deciduous teeth
BOHLAT-P: Oral Health Literacy Assessment Task for Paediatric Dentistry
