# Supplementary material for: Influence of digital media in the oral health education of mother-child pairs: study protocol of a parallel double-blind randomized clinical trial
Source: Trials. 2022 Aug 9;23:639. doi: 10.1186/s13063-022-06602-4 (PMC9361624; doi:10.1186/s13063-022-06602-4)

**UNIVERSITY OF SAO PAULO**

**SCHOOL OF DENTISTRY OF RIBEIRÃO PRETO**

**PÓS GRADUATE PROGRAM**

**DEPARTMENT OF PEDIATRIC DENTISTRY**

**LITERACY ASSESSMENT INSTRUMENT - BOHLAT-P**

## Dear Mother

First, thank you for agreeing to participate in our study!
This study is being carried out to better understand the familiarity of parents/guardians with words and dental information. responding to following questions will help us improve oral health communication between professionals and patients.

## PLEASE REMEMBER:

There are no right or wrong answers;
Be as sincere as possible. Answer the questions yourself and not consult other sources of information;
Your answers are confidential, no one will see them;
Some parts of the questionnaire have supporting text. Reading these texts it is essential for you to answer the questions.

Name: ____________________________________________________________Date:__/__/__

Child's name: ___________________________________________________________________________

**Part 1**

**Instructions**: Match the name and the picture by putting the correct number in the box (**<u>One answer only per box</u>**).

1.

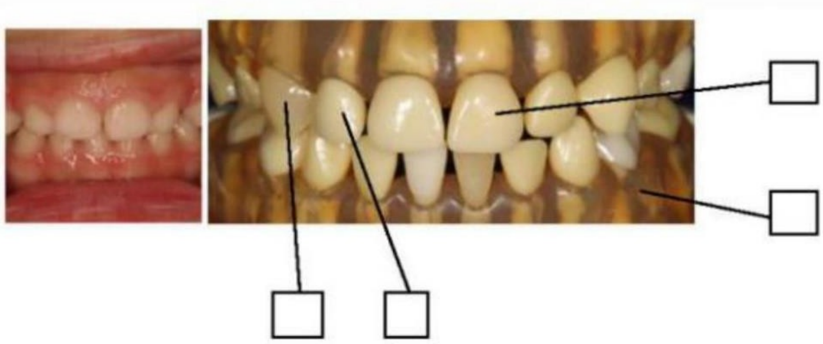

1. Canine
2. Palate
3. Lateral Incisor
4. Central Incisor
5. Molars
6. Gum

2.

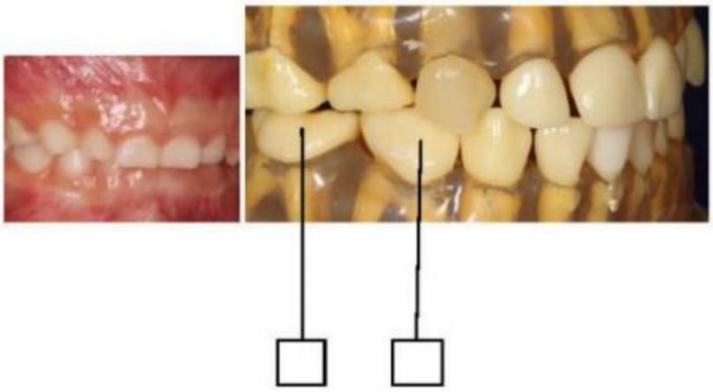

1. First Molar
2. Gingiva
3. Second Molar
4. Lips

**Part 1**

Instructions: Match the name and the picture by putting the correct number in the box (**One answer only per box**).

3.

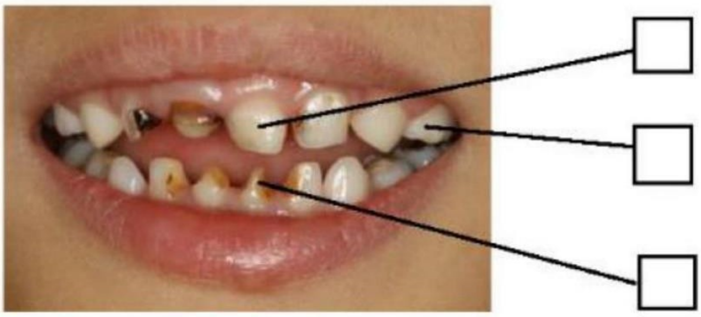

1. Central incisor
2. Calculus
3. First Molar
4. Caries
5. Amalgam

4.

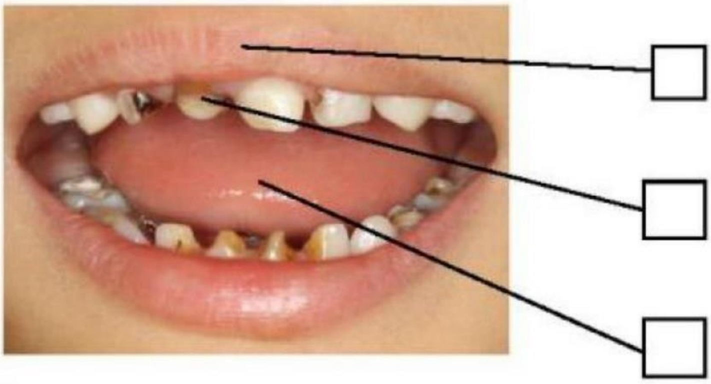

1. Lip
2. Denture
3. Tongue
4. Caries
5. Molars

**Part 2**

**Instructions**: Use the information and instructions after a dental consultation to answer the questions below.

1. Clinic Appointment Card

THE PRINCE PHILLIP DENTAL HOSPITAL

34 Hospital Road, São Paulo, Brazil

MON-FRI 8:30-5:30(excluding public holidays)
Tel:28590257

**APPOINTMENT CARD**

Record No ……………..
DEPARTMENT OF CHILDREN'S
DENTISTRY & ORTHODONTICS

NAME: José Aparecido Gomes
ADDRESS:________________________________

ALWAYS BRING YOUR CARD WITH YOU
WHEN ATTEND FOR AN APPOINTMENT

If you are unable to keep an appointment,
Please let the Hospital know by telephone.

Please be punctual for your appointment.

Every effort will be made to avoid keeping
You waiting but this cannot be guaranteed.

| DATE | TIME | OPERATOR | DATE | TIME | OPERATOR |
|---|---|---|---|---|---|
| June 1 | 9:45 a.m. | | | | |
| | | | | | |
| | | | | | |
| | | | | | |
| | | | | | |

**Part 2**

Q1: According to the information on the previous page, when will the next your child's consultation?

Answer:____________________________________________________

Q2: Does this mean your child must leave the house at 9:45 am?

Answer:____________________________________________________

Q3: Which number should you call if you need to cancel the appointment from your son?

Answer:____________________________________________________

Q4: What do you need to bring to your child's appointment?

Answer:____________________________________________________

**Part 2**

**Instructions**: Use the information and instructions after a dental consultation to answer the questions below.

2. Prescription Label

Q1: When is the expiry date of the medication?

A: ______________________

Q2: If your child takes the first dose of syrup on Friday at 8 a.m., when should your child take the next one?

A: ______________________

Q3: If your child's symptoms are gone by the 3rd day of taking medication, should you stop giving him/her this medication?

A: □ Yes □ No

Q4: This is an "antibiotic". How does an antibiotic help cure diseases?

A: □ Kills germs
□ Stops bleeding
□ Stops fever

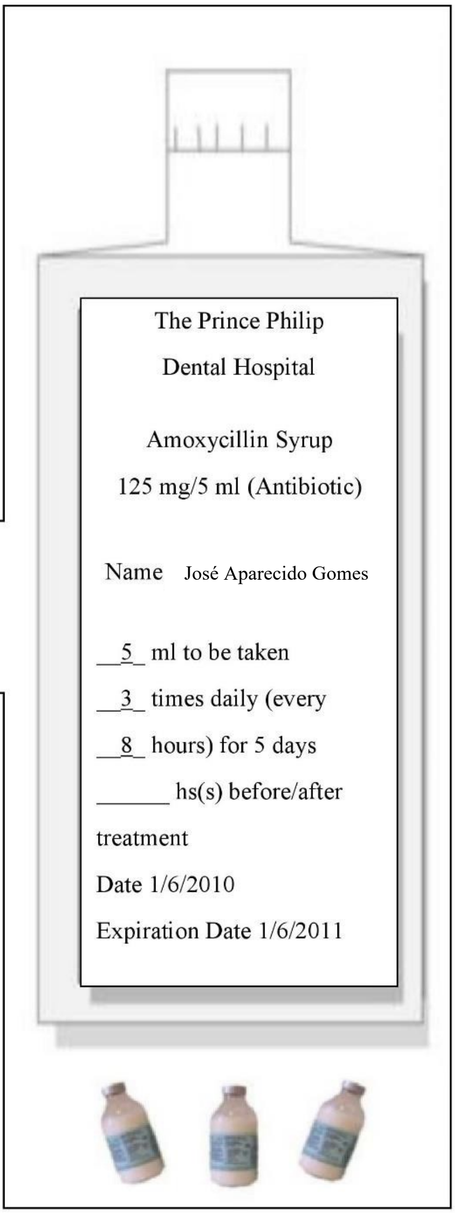

3. Prescription Label

The Prince Philip Dental Hospital

Name José Aparecido Gomes

__1__ tablets to be taken ______times daily

(every __4__ hours)

□ Before meal □ After meal

□ in the morning □ at bed time

□ to be chewed □ to be sucked

□ for pain ☒ when required

□ mouthwash □ local application

□ ___ hours before treatment

___ times daily

□ ___ hours after treatment

Panadol

250 mg

Store in a cool (below 25° C)
dry place
protect from light
keep out of reach of children

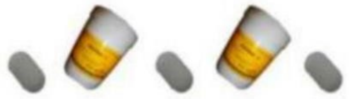

Q1: If your child is not feeling any pain, should your child take the medication?

A: □ Yes □ No

Q2: Can you take this medicine on July 2011?

A: □ Yes □ No

Q3: If your child is taking this drug, how many capsules can your child take per day?

A: ______________________

**Part 2**

**Instructions**: Use the information and instructions after a dental consultation to answer the questions below.

4. Post-Operative Instructions

Post-Operative Instructions
Paediatric Dentistry & Orthodontics

The Prince Philip Dental Hospital

1. Do not allow your child to take hot food or hot drinks for 4 hours after the operation of tooth extraction.
2. Do not allow your children to engage in strenuous exercise on the day of the operation or tooth extraction.
3. On the day after the operation or tooth extraction, your child can start using warm salt mouth washes. This should be done frequently to keep the socket or wound clean.
4. If a bleeding re-commences:
    a. The mouth should be rinsed with cold water once, then fold a piece of gauze (or a cotton handkerchief) into a tight pad and place directly over the bleeding area. Apply firm steady pressure on the pad by getting your child to bite for 15 minutes.
    b. This procedure should be repeated twice if bleeding persists. If this fails, telephone this Hospital* for professional advice.
5. For relief of pain, your child should take, every 6 hours, the tablets or syrup that was supplied If pain increases during the post-operative period, telephone this Hospital* for advice.
6. Some facial swelling is to be expected after surgery in mouth. There is no need for alarm. The swelling will subside in 3 to 4 days.
7. Following the use of local anaesthesia, the lips, tongue or cheeks may remain numb for 2 to 3 hours, during which time they may be damaged by biting. Young children especially should be reminded about harming themselves in this way.

*Post-Operative Emergencies
Prince Philip Dental Hospital
Monday to Friday: 8:30 a.m. to 5.00 p.m. (except public holidays)
Tel: 28590257 or 28590386

After office hours: Tel: 28590238

June 1, 1992

Q1: If the operation finishes at 2 p.m., when is the earliest that your child can have hoot foods or drinks?

A: ______________________________________________

Q2: (i) When do you start salt washing?

A: ______________________________________________

(ii) Why do you need to do this?

A: ______________________________________________

Q3: What should you do if your child's wound starts bleeding?

A: ______________________________________________

Q4: If bleeding persists, where should you get professional advice?

A: □ Queen Mary Hospital □ Prince Philip Dental Hospital

□ Queen Elisabeth Hospital □ Duchess of Kent Children's Hospital

Q5: What is the first step in pain relief?

A: ______________________________________________

Q6: If the surgery took place on Monday, when the swelling subside?

A: ______________________________________________

Q7: Why does your child needed to be observed carefully for 2-3 hours after the operation?

A: ______________________________________________

**Part 2**

**Instructions**: Use the information and instructions after a dental consultation to answer the questions below.

5. Toothpaste Tube

Q1: According to the toothpaste tube label below, should your child brush his/her teeth after breakfast?

A: □ Yes □ No

Q2: If your child is 1 ½ years old, can you brush his/her teeth with this toothpaste?

A: □ Yes □ No

Q3: What is the minimum number of times your child should brush his/her teeth each day?

A:____________________________________

Q4: if your child is taking fluoride supplements, what should you do if you want to use this toothpaste?

A:____________________________________

Q5: Which of these pictures best matches the amount of toothpaste that should be used for a child under 6 years of age? (Put a tick in the box).

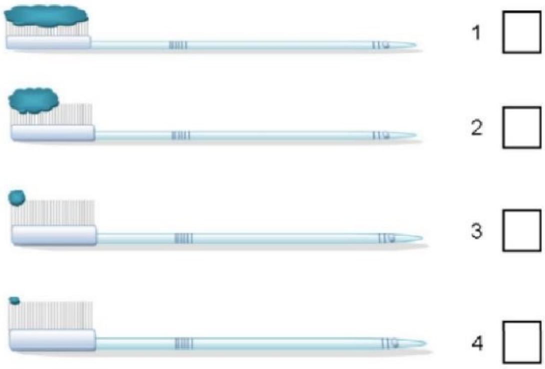


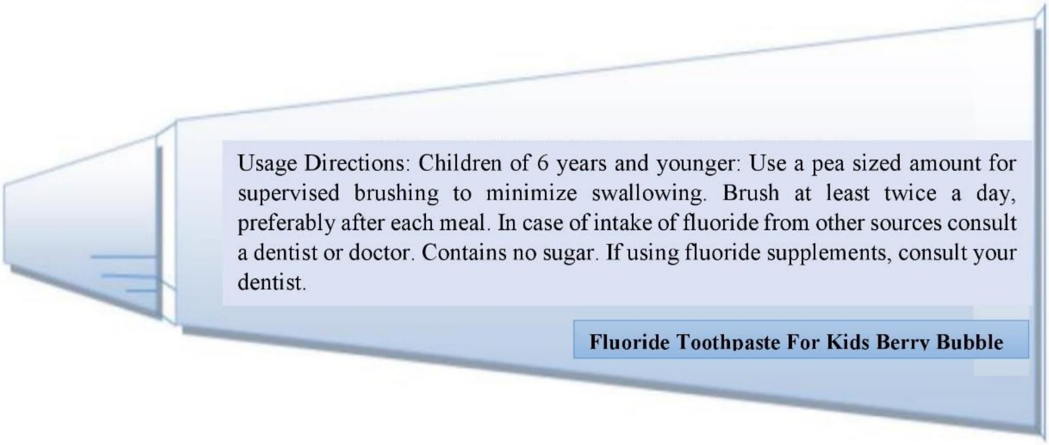

**Part 3**

**Instructions**: From the list provided, choose the best words to complete the advice from the dentist.

1. Parent Forum on Children's Tooth Development

| | |
|---|---|
| Parent's Question: | **What is tooth transition?** |
| Dentist's Answer: | People have two sets of teeth in a lifetime. Primary teeth are the first set of teeth we have and there are altogether 20 of them. During the tooth transition period, the primary teeth will begin to wiggle. They will eventually shed ____________ and subsequently the permanent teeth underneath will in<br>1. unscathed<br>2. disorderly<br>3. one by one<br>4. randomly<br><br>turn erupt into their spaces. Tooth transition period normally lasts form the age of 6 until approximately the age of 12. |
| Parent's Question: | **My child's central incisor are crowded, do we need to find a dentist to remove adjacent baby teeth?** |
| Dentist's Answer: | Since the permanent teeth are ________than the deciduous teeth, and if the _______ growth has not<br>1. fatter<br>2. smaller<br>3. larger<br>4. thinner<br><br>1. Jaw<br>2. gingival<br>3. teeth<br>4. lips<br><br>caught up with the eruption of the ________ teeth, there will be not enough room for straight<br>1. gum<br>2. caries<br>3. deciduous<br>4. permanent<br><br>alignment and the permanent teeth will be crowded out. We can determine if true crowding exists only after the permanent ___________ have erupted and the growth of jaw bones is ___________.<br>1. premolars<br>2. overlapping teeth<br>3. molars<br>4. wisdom teeth<br><br>1. approval<br>2. stabilized<br>3. deciduous<br>4. nutrition |
| Parent's Question: | **If there is a gap between the two upper permanent incisors when they erupt and they seem to be flared, does my child need braces?** |
| Dentist's Answer: | Some dentists refer to this condition as the "ugly sucking stage". This is only a ___________ stage.<br>1. transitional<br>2. late<br>3. permanent<br>4. early<br><br>Under normal circumstances, as the jaw matures and _______ and the canines erupt, the__________<br>1. open<br>2. close<br>3. grows<br>4. maintain<br><br>1. premolars<br>2. molars<br>3. root<br>4. incisors<br><br>will straighten up and the gap will be closed. |

**Part 3**

**Instructions**: The guide below has been mixed up. Label the boxes on the diagram to re-arrange the sentences in their correct order. The first one has been completed for you.

2. Tooth Brushing Guide

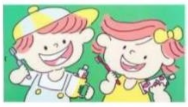

To effectively clean your child's teeth, it is important to use a correct tooth brushing method.

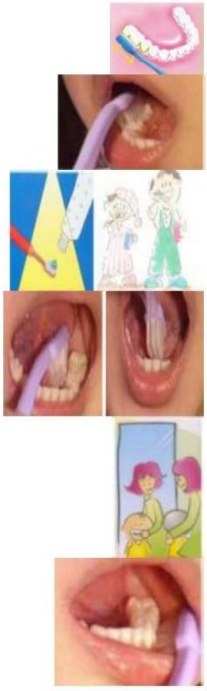

A. When you start brushing, work on the two or three teeth at a time, gently brush in a small scrubbing motion fat at least ten times in the following sequences:

B. Finally, brush the chewing surfaces of all teeth.

C. Let your child brush his/her own teeth every morning and before bed at night.

D. Then brush the inner surfaces of the upper and lower teeth.

E. Closing tip: Allow your child to try brushing on his/her own first. Then finish off by brushing your child's teeth thoroughly for him/her.

F. Firstly, the outer surfaces of the upper and lower teeth.

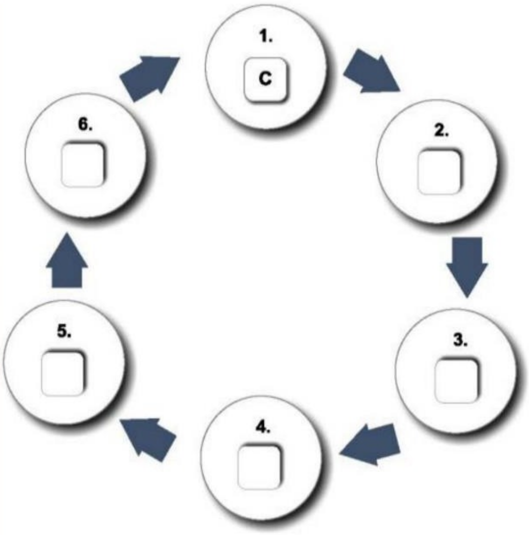

Supplement: Supplementary file 1 — Additional file 1. [file 13063_2022_6602_MOESM1_ESM.pdf]
